# Editorial: Antimicrobial resistance and stewardship in the COVID-19 era

**DOI:** 10.3389/fpubh.2024.1398981

**Published:** 2024-05-01

**Authors:** Ashish Kumar Kakkar, Nusrat Shafiq

**Affiliations:** Department of Pharmacology, Post Graduate Institute of Medical Education and Research (PGIMER), Chandigarh, India

**Keywords:** COVID-19, antimicrobial resistance (AMR), antimicrobial stewardship (AMS), SARS- CoV-2, antimicrobial consumption

Since its onset in December 2019, the COVID-19 pandemic has led to unprecedented disruptions across the global economy, healthcare systems, and societal infrastructure, causing significant loss of life and widespread morbidity. Four years on, as per WHO estimates, globally more than 774 million cases of COVID-19 have been reported while the total number of deaths stands in excess of 7 million (1). Several studies, both from developed as well as developing country settings have indicated that the usage of antimicrobials increased during the COVID-19 pandemic fueled by the concerns regarding bacterial co-infections and secondary infections among those affected pointing toward a pattern of unwarranted and excessive prescribing (2, 3). At the same time, the pre pandemic antimicrobial resistance (AMR) surveillance and antimicrobial stewardship activities were significantly curtailed owing to the reallocation of limited health system resources toward emergency response measures. The Centers for Disease Control and Prevention (CDC) report on COVID-19 impact on antimicrobial resistance concluded that the threat of multidrug resistant infections got worse during the pandemic associated with increased antimicrobial use, availability of fewer AMR data and reduced comprehensive prevention practices (4). With this background, the goal of the Research Topic “*Antimicrobial resistance and stewardship in the COVID-19 era*” was to explore the impact of the COVID-19 pandemic on healthcare and social care services focusing specifically on the effects on antibiotic use and resistance and examine the influence on antimicrobial stewardship (AMS) activities (5).

The systematic review by Fukushige et al. evaluated the impact of the pandemic on antimicrobial consumption specifically comparing the metrics between 2019 and 2020. The authors reported consistent reductions in the community antimicrobial consumption across various studies while the results from hospital-based studies were more variable with studies showing both increased as well as decreased usage. Importantly, however, studies that reported interruptions of the AMS activities found increased antibiotic consumption in 2020 when compared to data from 2019. The authors also highlighted the lack of availability of relevant data from several low- and middle income countries that impeded accurate estimation of impact of pandemic on the antimicrobial consumption in these territories. The article by Butt et al. discusses the alarming rise in Extensively Drug-Resistant Typhoid Fever (XDR-TF) cases in Pakistan, posing a significant public health threat and potentially marking a regression to the pre-antibiotic era. It highlights the challenges in controlling the spread due to inadequate sanitation, water contamination, and the indiscriminate use of antibiotics. The introduction of the Typhoid Conjugate Vaccine (TCV) into Pakistan's immunization program represents a crucial step toward combating this issue. However, the article emphasizes the need for comprehensive strategies, including improved sanitation, water quality, and vaccination campaigns, to effectively address the XDR-TF crisis and prevent a public health catastrophe. Importantly, the authors highlight that excessive use of azithromycin for COVID-19 treatment could have compromised one of the limited effective options against Extensively Drug-Resistant *Salmonella* Typhi raising concerns regarding the potential for increased AMR.

The opinion paper by Kanj et al. highlights the value of continued AMS activities during the pandemic. The authors argue that COVID19 pandemic has revealed significant vulnerabilities in global health systems, particularly affecting AMS programs and exacerbating the threat of antimicrobial resistance. This situation necessitates enhanced global coordination and strengthening of AMS programs, improved diagnostic access, and the dissemination of guidelines for appropriate antimicrobial use. The pandemic has led to increased inappropriate prescribing of antibiotics for COVID-19 patients, further risking AMR. The paper advocates for comprehensive strategies integrating AMS, infection prevention, control, and microbial surveillance to combat AMR effectively amidst ongoing and future health crises.

The increased use of antimicrobials in the initial phase of the COVID-19 pandemic was driven largely by fear of secondary bacterial infections especially in the critically ill or mechanically ventilated patients. Several international and national guidelines have emphasized the importance of rational antimicrobial use in the management of COVID-19. The World Health Organization, the Infectious Diseases Society of America amongst others have recommended against the routine use of antibiotics in COVID-19 patients unless there is clinical suspicion or confirmation of a bacterial or fungal co-infection (6, 7). In our own experience, antimicrobial stewardship practices at our institute had to be adapted including the integration of telemedicine and remote consultations to facilitate development of COVID-19 management guidelines, education of healthcare professionals as well as audit and feedback of antimicrobial prescriptions (8).

The COVID-19 pandemic has highlighted the delicate balance between the need for antimicrobials in treating secondary infections and the risk of exacerbating antimicrobial resistance. The increased use of antibiotics during the pandemic, particularly in resource limited settings with limited diagnostic capabilities, poses a risk for the acceleration of AMR. This makes the role of AMS programs crucial in monitoring antimicrobial use, promoting adherence to treatment guidelines, and ensuring the rational use of antibiotics during and beyond the pandemic. The COVID-19 pandemic highlights the need for robust antimicrobial stewardship practices as an integral part of pandemic preparedness and response. It underscores the importance of rapid diagnostic tests, rational use of antimicrobials, and the continuous education of healthcare professionals on antimicrobial stewardship principles. Addressing the challenges posed by the pandemic requires a coordinated effort among various stakeholders - clinicians, clinical pharmacologists, microbiologists, pharmacists, and public health professionals to optimize antimicrobial use and mitigate the risks of antimicrobial resistance.

During the worst phases of the COVID-19 pandemic, tackling AMR was seen as a problem left best for the future and those of us living in that post-pandemic future should consider ourselves lucky. However, the damage inflicted to the AMR containment efforts by the indiscriminate use of antimicrobials during COVID-19 especially in the hospital settings needs to be undone now by continuing and building upon the pre-pandemic efforts and getting back to the basics: tracking AMR, preventing infections in the first place, judicious use of antimicrobials based on indication, choice of agent, dosage and duration, and spreading education and awareness regarding AMR among healthcare professionals as well as general public.

## Author contributions

AK: Conceptualization, Data curation, Writing – original draft. NS: Conceptualization, Supervision, Writing – review & editing.
